# Commissioning of a commercial treatment planning system for scanned carbon‐ion radiotherapy

**DOI:** 10.1002/acm2.14580

**Published:** 2024-11-29

**Authors:** Wei Sun, Weiwei Wang, Zhijie Huang, Jingfang Zhao

**Affiliations:** ^1^ Department of Medical Physics Shanghai Proton and Heavy Ion Center Shanghai Key Laboratory of Radiation Oncology Shanghai Engineering Research Center of Proton and Heavy Ion Radiation Therapy Shanghai China; ^2^ Department of Medical Physics Shanghai Proton and Heavy Ion Center Fudan University Cancer Hospital Shanghai China; ^3^ Institute of Modern Physics Applied Ion Beam Physics Laboratory Fudan University Shanghai China

**Keywords:** carbon ions, pencil beam scanning, TPS commissioning

## Abstract

**Purpose:**

To commission the RayStation (RS) TPS (treatment planning system) for scanned CIRT (carbon‐ion radiotherapy) utilizing pencil beam algorithms (PBv4.2).

**Methods:**

The beam model commissioning entailed employing 1D single beams and 2D monoenergetic fields to validate spot profiles with films, assess beam range using Peakfinder measurements, and evaluate fragment spectra through dose‐averaged linear energy transfer (LETd) calculations. 3D dose distributions were verified in homogeneous phantoms for both absorbed and relative biological effectiveness (RBE)‐weighted doses, and further assessed in double wedge and anthropomorphic phantoms for absorbed dose only. Finally, RBE‐weighted dose verification and patient‐specific quality assurance were conducted using 58 beams from 20 clinically treated patient plans.

**Results:**

The results demonstrated good agreement in absolute dose distribution between TPS calculations and measurements, with mean dose discrepancies within 3%. However, deviations were slightly higher (> 1%) for the cases involving the range shifter (RaShi) compared to those without the RaShi (< 1%). Beam range, depth dose distribution, and lateral profiles of spread‐out Bragg peaks (SOBPs) closely matched between RS TPS calculations and measurements. Some discrepancies (less than 0.5 mm) were observed at field edges and in penumbra regions due to limitations in simulating asymmetrical spots, but within clinical tolerance. After model tuning, RBE‐weighted dose calculations in RS TPS were in agreement with those from the clinically used TPS, except for variations exceeding 3% observed at energies exceeding 408.07 MeV/u, primarily attributed to fragment spectra differences.

**Conclusion:**

Overall, this study validated the RS TPS for calculating absorbed doses against measurements and RBE‐weighted doses against a clinically used TPS. The results suggested that the RS TPS could be utilized for CIRT treatment planning, except for energies exceeding 408.07 MeV/u.

## 1 INTRODUCTION

Carbon‐ion radiotherapy (CIRT) stands out for its substantial physical and biological advantages over photon radiotherapy, establishing itself as a crucial treatment modality in external beam radiotherapy. The treatment planning system (TPS) plays a pivotal role in this process by optimizing treatment plans and converting the radiation oncologist's prescription into a deliverable dose,^1^ thereby harnessing the unique benefits of CIRT.

Prior to clinical implementation, thorough commissioning of the TPS is essential to ensure precise dose calculations. The distinct physical and biological properties of carbon‐ion beams render the commissioning of a CIRT TPS inherently more complex than that of photon and proton therapies.

The RayStation (RS) TPS [version 10B, RaySearch Laboratories (RSL), Stockholm, Sweden] stands among the few commercially available TPSs for CIRT. While extensive studies have been conducted on TPS commissioning for proton therapy,^2^, ^3^, ^4^, ^5^ research on CIRT has primarily focused on specific topics such as the accuracy of pencil beam dose calculation in heterogeneous geometries,^6^ benchmarking linear energy transfer (LET) with the Monte Carlo (MC) algorithm,^7^ and assessing the impact of particle energy spectra on relative biological effectiveness (RBE)‐weighted dose calculation.^8^ A single report has addressed the commissioning of the VQA Plan TPS employing the microdosimetric kinetic model (MKM) and physical beam model for scanned carbon‐ion beams.^9^ However, given that absorbed and RBE‐weighted dose calculation algorithms may vary across TPSs, a comprehensive commissioning process for CIRT in RS TPS, incorporating the utilization of the local effect model (LEM) and dose algorithms different from the VQA Plan TPS, remains unreported to date.

In contrast to proton therapy, carbon ions undergo more extensive nuclear reactions that significantly influence dose distribution, resulting in a distal tail and a broader, low‐intensity distribution at the lateral side, often called the “nuclear halo.” In CIRT treatment planning, pencil beam algorithms (PBAs) are predominantly used due to their rapid calculation speed and efficiency. Despite their less accurate description of ion transport and interactions within the beamline and target, correction models incorporating additional wider Gaussians,^10^ as well as other more complex functions,^11^, ^12^, ^13^ are utilized to address inherent limitations in accounting for nuclear reactions.^14^ Therefore, validation beyond the target region is also essential, encompassing the physics of the distal tail, penumbra, and out‐of‐field areas. Additionally, the performance of PBAs should be assessed in heterogeneous situations.

Furthermore, in CIRT treatment planning, optimizing the RBE‐weighted dose for clinical cases necessitates consideration of various factors, including dose‐averaged linear energy transfer (LETd), fragment spectra, and absorbed doses. The CIRT TPS commissioning should thoroughly incorporate these considerations. The RBE‐weighted dose in RS TPS should be aligned with the clinically used dose calculated by Syngo RT planning TPS (Syngo TPS), which has successfully treated over 6000 patients in our facility.

This study focuses on commissioning the RS TPS for CIRT, with the aim of accurately calculating absorbed and RBE‐weighted doses, ensuring clinical outcomes aligned with existing protocols. The study encompasses the essential steps involved in configuring and validating the new TPS for CIRT, providing valuable insights for other medical centers undertaking similar endeavors.

## 2 METHODS

### 2.1 Overview

This study employed the RS TPS V10B, incorporating PBAs (version 4.2) tailored for carbon‐ion beams. The study comprised two main phases: the initial construction of the beam model in alignment with RSL specifications and subsequent validation. Validation involved three critical components: (i) the commissioning of a physical beam model, evaluating its capability to replicate characteristics of a single static pencil beam; (ii) commissioning of 3D dose calculations, assessing the accuracy of calculations for 3D treatment plans; and (iii) patient case verification, aimed at confirming the absorbed and RBE‐weighted dose calculation accuracy for clinical patients. For comprehensive details on commissioning tests and equipment, please refer to Supplement 1. All ionization chambers used in this study were calibrated against a Farmer chamber calibrated by an Accredited Dosimetry Calibration Laboratory.

### 2.2 Beam model generation

In accordance with the modeling prerequisites for a scanned carbon‐ion beam delivery system, the following sections outline the key components of the dataset submitted to the RSL for the generation of the beam model.

The integrated depth dose distribution (IDD) measurements were performed for 26 out of 291 carbon energies with a PEAKFINDER (type 41030; PTW, Freiburg, Germany). It was ensured that there was a water equivalent gap of less than 2.0 cm between any two neighboring energies. These measured IDDs served as the basis for interpolation to derive depth dose distributions (DDDs) for all energies required for beam modeling. Additionally, the correction factor for the finite volume of the Bragg peak chamber (type 34080; PTW, Freiburg, Germany) was determined by the RSL.

The full‐widths at half maximum (FWHMs) of 37 energies in the air were measured using a multiple wire proportional counter (MWPC). Measurements were conducted in the air for each energy at seven positions along the central axis of the beam line. Spot profiles were then created as Gaussian distributions using the measured FWHMs. There are five levels of the spot size in our particle therapy system, i.e., IONTRIS system (Siemens, München, Germany). The RS TPS does not allow arbitrary selection and definition of focus levels in the model. Therefore, the spot data collected here were limited to the most commonly used level.

The absolute dosimetry (AD) in water was calibrated in centi‐gray per particle number (cGy/NP) for sixteen energy levels, calculated as:

(1) $$\begin{array}{cc} AD\;in\;\frac{cGy}{NP} & =\frac{D_{m}\;\left[cGy\right]}{Total\;number\;of\;particles\;\left[NP\right]}\\ & =\frac{D_{m}\;\left[cGy\right]}{NP\;per\;spot\times N_{spot}\times N_{scan}} \end{array}$$

where $D_{m}$, $N_{spot}$ and $N_{scan}$ are measured dose, number of spots and number of scans, respectively. Measurements were performed at a depth midway between 1 cm and one half of the position of the Bragg peak maximum. This was accomplished using a single scanning field of 10.0 × 10.0 cm^2^ and a spot spacing of 0.25 cm. This method was chosen due to the limitations^15^ of the IAEA TRS‐398.^16^ protocol. Furthermore, it was recommended by the RS user manual and supported by previous studies.^17^, ^18^, ^19^

The fragment spectra, utilized in the calculation of RBE‐weighted dose, were simulated using MC FLUKA^11^ and has been applied in clinical treatment planning for seven years. To align with the specifications of the RS TPS, the fragment spectra underwent reformatting and integration by RSL.

### 2.3 1D/2D Beam model commissioning

#### 2.3.1 Lateral spot profiles in air

The lateral spot profiles of five energies were measured with extended dose range (EDR2) films (Carestream, NY, USA), which were calibrated for carbon‐ion dose measurements prior to this study,^20^ put at five positions along the central axis: the isocenter (86.5 cm downstream the nozzle), 80 and 40 cm upstream the isocenter, 20 and 60 cm downstream the isocenter. Profiles and FWHM in X‐ and Y‐directions of each spot were automatically generated across the spot center using FilmAnalyze software (PTW, Freiburg, Germany). Sigma was derived as $FWHM/(2\sqrt{2\ln2})$, assuming that the spot is Gaussian. The dose deposition generated by the TPS at the same air position were simulated to verify against the corresponding measurements. Spot sigmas and profiles were then compared between measurements and simulations.

#### 2.3.2 Beam range in water

The TPS generated DDDs were computed within a virtual cuboid‐shaped water phantom (WP) along the central axis. Twenty‐six monoenergetic beam plans were created in the WP, each featuring a field size of 10.0 × 10.0 cm^2^, spot sizes ranging from the FWHM of 0.8 to 1.0 cm, and the spot spacing of 0.25 cm. The calculated DDDs were compared to the measured IDDs regarding the beam range R_80_, i.e., the range of the distal 80% of the maximum dose. The IDD was measured at the exit of the beam application and monitor system (BAMS). During comparison, the additional water equivalent length (WEL) of the air gap from the BAMS to the isocenter was taken into account.

#### 2.3.3 Fragment spectra verification

Fragment spectra of carbon‐ion beams with atomic numbers ranging from 1 to typically 6, which are in agreement with our clinical Syngo TPS, are integral for calculating RBE‐weighted doses and serve as the baseline for RS TPS. To assess the accuracy of the fragment spectra, the LETd was calculated using a methodology derived from previous studies.^21^ A comparison was then made between the LETd values obtained from our newly configured TPS and those calculated using the baseline data from the Syngo TPS. The fragment spectra of selected nominal energy in Syngo TPS were processed by a C++ script into Excel files and the LETd was calculated using the fragment spectra data according to the method published.^22^ The analysis encompassed five monoenergetic carbon‐ion beams with energies of 140.01, 219.20, 318.79, 408.07, and 428.38 MeV/u in a water medium.

### 2.4 3D dosimetric commissioning

#### 2.4.1 Absolute dose verification in spread‐out Bragg peaks (SOBPs)

The absolute dose was verified by comparing RS TPS calculation results with the average of 10 independent measurements from seven SOBP plans in a WP over the past five years. The seven plans with cubic targets of size 8.0 × 8.0 × 8.0 cm^3^ were initially created and optimized in clinical used Syngo TPS. For this study, these plans were imported into RS TPS, and the dose distribution was recalculated. Within the WP, five cubes were centered at 6.5 cm, 10.0 cm, 15.0 cm, 20.0 cm, and 25.0 cm downstream from the surface, while two additional cubes were centered at 6.5 cm with the range shifter (Rashi), with air gap of 20 and 30 cm from the RaShi to the front surface of the WP. The target median dose was 2.0 Gy.

Measurements were performed using a 3D dosimetric verification system^23^ with a 24‐PinPoint chamber (PPC) array in an MP3‐P WP (PTW, Freiburg, Germany). The 24‐PPC array is comprised of 24 pin‐point ionization chambers (ICs) (TM31015, PTW, Freiburg, Germany) and a 3D polymethyl methacrylate (PMMA) detector block which holds the chambers and arranges them in different rows and lines to prevent shadowing each other. This setup enables simultaneous measurements with all 24 chambers and facilitates various positions in MP3‐P WP. The mean local difference of each plan between TPS calculated and measured doses was calculated as follows:

(2) $$\Delta \;{\bar{D}}_{L}=\frac{1}{24}\;\cdot \sum_{i=1}^{24}\frac{D_{TPS}^{i}-D_{meas}^{i}}{D_{meas}^{i}}\times 100\%$$

where $D_{TPS}^{i}$ is the TPS calculated dose, and $D_{meas}^{i}$ is the average measured dose of IC *i*.

Based on the dose differences of SOBP plans, the beam model was fine‐tuned by adjusting the absolute output calibration factors (AOCFs) to reduce $\Delta {\bar{D}}_{L}$ to within ± 1%.

#### 2.4.2 Depth dose comparisons of SOBPs in homogeneous condition

Five plans with 10.0 × 10.0 × 5.0 cm^3^ cuboid targets, centered at 5.0 cm, 10.0 cm, 15.0 cm, 20.0 cm, and 27.5 cm depth, were created and optimized in RS TPS. The median dose was 1.0 Gy. Measurements were carried out in the MP3‐P WP with the 24‐PPC array at multiple steps to acquire the DDDs along the central axis of the SOBP. The mean global deviation of absorbed dose of each beam between RS TPS calculation and measurement was calculated as:

(3) $$\Delta \;{\bar{D}}_{G}=\frac{1}{N}\;\cdot \sum_{i=1}^{N}\frac{D_{TPS}^{i}-D_{meas}^{i}}{D_{max}}\times 100\%$$

where *N* is the number of ICs in specific regions of interest, namely, the entrance (proximal dose < 95% of the prescribed dose, PD), target (dose ≥ 95% PD), distal‐fall‐off (DFO, where the dose gradient greater than 0.5 Gy/cm beyond the SOBP), and tail (where the dose gradient smaller than 0.5 Gy/cm beyond the SOBP) regions. Those four regions were analyzed separately. $D_{max}$ is the maximum measured dose of the whole depth dose curve. The distal edge of depth dose was evaluated by R_80_.

#### 2.4.3 Comparisons of lateral profiles for different field sizes

RS TPS calculated lateral profiles were compared against measurements using a 2D detector array (OCTAVIUS Detector 729 XDR; PTW, Freiburg, Germany) for 16 treatment plans. Twelve of these plans included four different cuboid target sizes (3.0 × 3.0 × 5.0 , 5.0 × 5.0 × 5.0 , 10.0 × 10.0 × 5.0 , and 15.0 × 15.0 × 5.0 cm^3^) centered at depths of 5.0 , 15.0 , and 27.5 cm. Additionally four plans incorporating the RaShi with an air gap of 30 cm were calculated with the targets centered at depth of 2.5 cm. A median dose of 1.0 Gy was prescribed to all plans.

During the measurement process, solid‐water phantoms with thicknesses of 5.0 , 15.0 , and 27.5 cm were positioned in front of the 2D detector array. Five repeated measurements were taken at different detector positions in the X direction with a step size of 0.2 cm. The results were then combined to generate a composite profile with a resolution of 0.2 cm. Each profile was normalized by the mean dose within the central 80% of the radiation field. Subsequently, these results were compared with the corresponding profile generated in the RS TPS.

#### 2.4.4 Absolute dose verification in shallow treatment targets

Five plans with different size of cuboid targets (3.0 × 3.0 × 3.0 , 5.0 × 5.0 × 3.0 , 10.0 × 10.0 × 3.0 , 15.0 × 15.0 × 3.0, and 19.0 × 19.0 × 3.0 cm^3^), centered at depth of 2.7 cm, were created and optimized in RS TPS. A median dose of 1.0 Gy was prescribed to each plan. The 24‐PPC array was used to measure the absolute dose within each target in the MP3‐P WP. The measurement results from PPCs inside the SOBPs were used for absolute dose comparison, including six PPCs for the 3.0 × 3.0 × 3.0 cm^3^ SOBP and 12 PPCs for each of the other four larger SOBPs.

#### 2.4.5 Comparisons in wedge and anthropomorphic phantom

A double‐wedge (DW) phantom and a half‐head (HH) anthropomorphic male Alderson Radiation Therapy (ART) phantom (Radiology Support Devices Inc., Long Beach, USA) were used to evaluate dose accuracy under oblique incidence and non‐homogeneous tissue.^24^ The phantoms were affixed to the front surface of the WP and pointed to the exit window of the nozzle. Regular shaped targets of various sizes (10.0 × 10.0 × 10.0 and 8.0 × 8.0 × 8.0 cm^3^) and centered at different depths (12.8  and 8.5 cm) in water were irradiated and measured with the 24‐PPC array. Measurements were carried out at different positions along the target's left‐right, superior‐inferior, and anterior‐posterior axes and categorized into different dose regions: the target, the out‐of‐field (dose<20% PD), and the penumbra region (20% ≤ doses ≤ 80% PD) for lateral direction; the entrance, the target, the DFO, and the tail region for longitudinal direction.

The CT scan of HH phantom was conducted with scanning parameters and calibration curve described in previous work.^25^, ^26^ The DW phantom was modeled as two wedges with PMMA material override. In TPS, the WP was modeled attached to the DW and HH phantoms as in the corresponding measurement.

The TPS calculated doses were compared to measurements regarding to each dose region. In addition, 1D gamma analysis^27^ (3%, 2 mm) was performed for the high gradient regions: the DFO and penumbra.

#### 2.4.6 Comparisons of RBE‐weighted dose in homogeneous phantom

The RBE‐weighted dose was calculated for the same plans as in 2.4.1 and compared between RS and Syngo TPS. Differences were analyzed longitudinally across all regions of interest, including entrance, target, DFO, and tail region. The RBE_max_ value table in the LEM model was fine‐tuned according to the original differences to make the RBE‐weighted dose in RS match the Syngo TPS in the same absorbed dose value.^28^ The Syngo TPS was chosen to be the reference of RS because its calculation has been proven accurate and safe in years of clinical application.

### 2.5 Patient case verification

#### 2.5.1 Validation of RBE‐weighted dose calculation

Twenty clinically treated patient plans, which were created in Syngo TPS and encompassed multiple sites such as head and neck, thorax, abdomen, pelvis, and extremities, were imported and recalculated in the RS TPS in the study. Those plans were all optimized in Syngo using intensity‐modulated particle therapy (IMPT) strategy. Fifty‐eight beams in total were included, of which 27 beams were without the RaShi and 31 beams with the RaShi. Beams with the RaShi had different air gaps from the patient external volumes, 28 having a 30 cm gap and three having a 20 cm gap. The overall disease characteristics of evaluated treatment plans are listed in Supplement 2.

The RBE‐weighted dose was computed in RS using the local effect model 1 (LEM1) with parameters identical to those used in LEM1 in Syngo. The LEM1 parameters are^29^: $\alpha =0.1\;Gy^{-1};\;\beta =0.05\;Gy^{-2};\;\;r_{n}=5\;\mu m;\;\;D_{t}=30\;Gy$. Where α,β are the linear and quadratic coefficients in the linear‐quadratic model describing cell survival after x‐ray exposure; $r_{n}$ is the radius of the cell nucleus which in our case is chordoma cell; and $D_{t}$ is the threshold dose where the cell survival curve transits from linear‐quadratic to purely linear shape. To compare the dose distributions between the two TPSs, we employed 3D gamma analysis with Verisoft software (Version 7.1; PTW, Freiburg, Germany). The gamma index criteria were set at 3 mm for distance‐to‐agreement (DTA) and 3% for dose difference, referenced to the PD. The criteria took consideration of the dose calculation grid 3 mm.

#### 2.5.2 Patient‐specific quality assurance

For the patient plans mentioned above, each beam has been measured using the 24‐PPC array in the MP3‐P WP in accordance with our routine PSQA procedure.^30^ TPS calculated dose corresponding to each PPC position was extracted using a python script in RS scripting interface. The mean global deviation of the absorbed dose for each beam was calculated using the results obtained from 24 PPCs' RS TPS calculations and measurements. Points were excluded from the evaluation if the dose was lower than 5% of the maximum calculated dose or the dose gradient divided by the maximum calculated dose exceeded 10%/mm. 1D gamma analysis (3%, 3 mm) was performed for the included dose points. The pass criteria for a beam were a mean global deviation ≤ 3% and a gamma pass rate ≥ 90%. These gamma analysis criteria were the same as what are clinically used in our facility.

## 3 RESULTS

### 3.1 1D/2D beam model commissioning

#### 3.1.1 Lateral spot profiles in air

**FIGURE 1:**
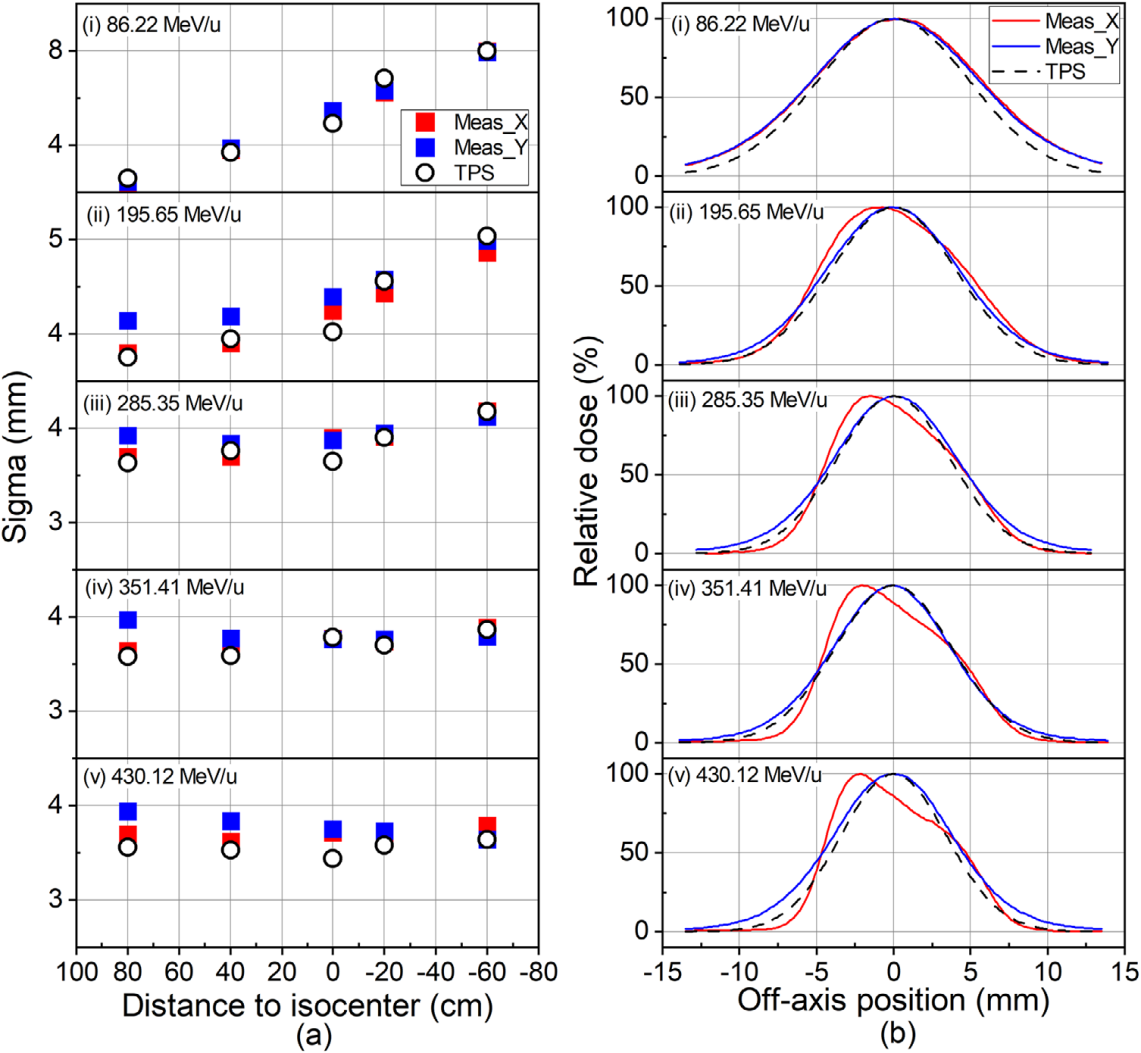
(a) The comparisons of RS calculated and film measured spot sizes in air in terms of sigma for five energies at different positions along the central axis. (i) 86.22 MeV/u; (ii) 195.65 MeV/u; (iii) 285.35 MeV/u; (iv) 351.41 MeV/u; (v) 430.12 MeV/u. (b) TPS‐calculated and film measured spot profiles for the same energies at isocenter.

TPS‐calculated and film measured spot sizes in terms of sigma for five energies are plotted in Figure 1a. The deviations between them range from −10.03% to 9.83%, a maximum of −10.03% for 86.22 MeV/u in the Y‐direction at the isocenter. TPS‐calculated and measured spot profiles at the isocenter are shown in Figure 1b.

#### 3.1.2 Beam range in water

**FIGURE 2:**
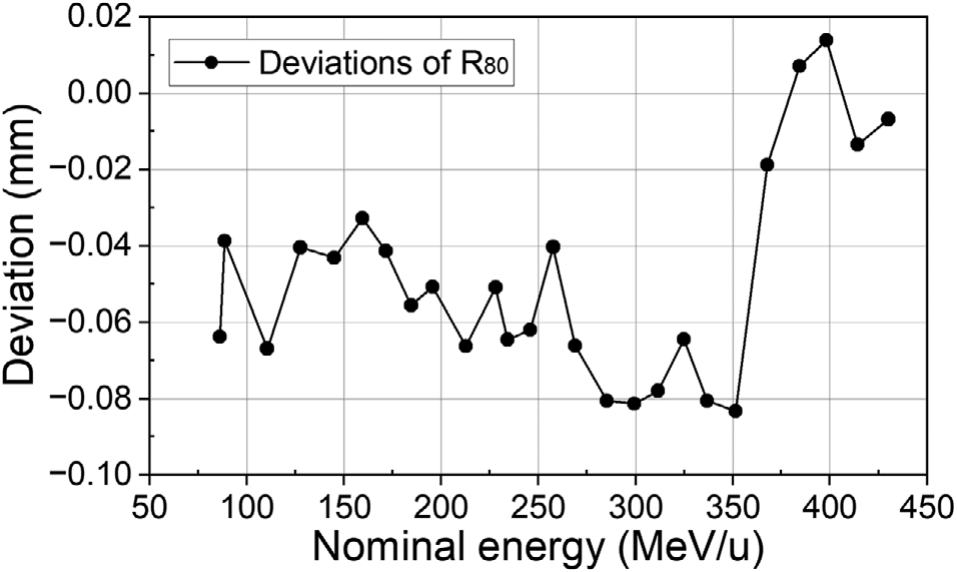
Deviations of *R*_80_ of 26 energies between RS calculations and measurements.

Deviations of *R*_80_ of 26 energies between TPS calculations and measurements are shown in Figure 2. The mean deviation between calculated and measured *R*_80_ of the 26 energies ranged from −0.08  to 0.01 mm.

#### 3.1.3 Fragment spectra verification

**FIGURE 3:**
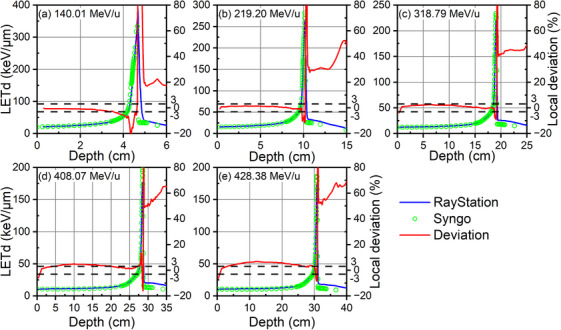
The comparisons of LETds of five monoenergetic carbon‐ion beams between RS and Syngo calculations.

In the analysis of five monoenergetic carbon‐ion beams with energies of 140.01, 219.20, 318.79, 408.07, and 428.38 MeV/u, LETd values were computed using both RS and Syngo baseline, as depicted in Figure 3 along with their differences. Prior to 408.07 MeV/u, the discrepancies of LETd between RS and Syngo remain within 3% for the entrance part. However, there is a shift occurred beyond this energy, where the deviation exceeds 3%, despite utilizing the same spectrum data in both RS and Syngo.

### 3.2 3D dosimetric commissioning

#### 3.2.1 Absolute dose verification in SOBPs

**FIGURE 4:**
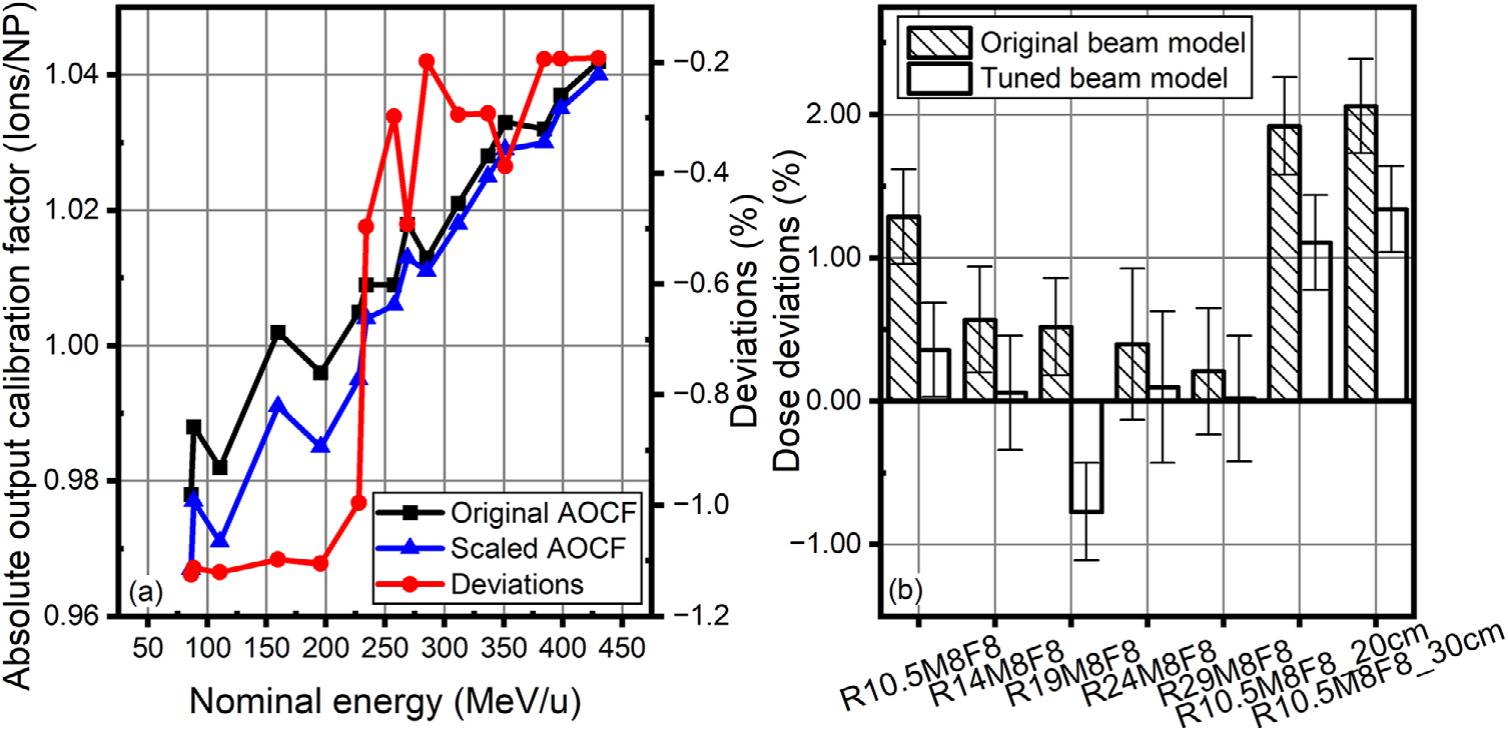
(a) The original and scaled AOCFs based on dose measurements in SOBPs. (b) Local dose deviations between RS calculations and measurements before and after beam model tuning in five SOBP plans without the RaShi: R10.5M8F8, R14M8F8, R19M8F8, R24M8F8, and R29M8F8, and two plans with the RaShi: R10.5M8F8_20 cm and R10.5M8F8_30 cm, where R stands for Range, M for Modulation width, and F for Field size. Plans are labeled according to their Range in cm, Modulation width (SOBP) in cm, square Field size in cm, and air gap distance in cm if a RaShi was used.

It shows in Figure 4b that the TPS overestimated the absolute outputs of low energies using the original AOCFs and beam model. Then, the beam model was tuned by reducing the AOCFs of corresponding energies by 0.2% ∼ 1.1% according to original measurement results. Calculated by the tuned beam model, the mean local dose differences from measurements were lower than 1% (maximum 0.77%) for the five SOBP plans without the RaShi, and still remained higher than 1% (1.11% ± 0.33% and 1.34% ± 0.30%) for two SOBP plans with the RaShi of 20 and 30 cm air gap.

#### 3.2.2 Comparisons of depth doses in homogeneous condition

The mean global dose deviations in four dose regions of five plans are presented in table 1. The mean global deviations are all within 3%. DFO regions with high gradient show higher deviations, the maximum point dose deviation reaching 5.34%, while points at other regions remain below 3%. The entrance dose of R7.5M5F10 was unavailable because the entrance was too shallow and the PPC array can only measure the dose deeper than 2.0 cm. The maximum difference of the *R*_80_ for five SOBP plans is 0.06 cm. The comparison plot of DDDs between calculated and measured values is provided in Supplement 3.

**TABLE 1:** Mean global dose deviations in four regions of five plans at different depths: R7.5M5F10, R12.5M5F10, R17.5M5F10, R22.5M5F10, and R30M5F10. Plans are labeled according to their range in cm, modulation width (SOBP) in cm, and square field size in cm.

	Mean global dose deviations (%)			
Plan	Entrance	SOBP	DFO	Tail
R7.5M5F10	N/A	1.45 ± 0.90	−0.69 ± 2.44	0.63 ± 0.62
R12.5M5F10	0.68 ± 0.23	0.99 ± 0.55	2.60 ± 1.88	0.33 ± 0.17
R17.5M5F10	0.13 ± 0.52	0.64 ± 0.47	1.42 ± 2.47	0.39 ± 0.25
R22.5M5F10	0.52 ± 0.53	0.84 ± 0.86	2.26 ± 1.68	0.12 ± 0.37
R30M5F10	0.30 ± 0.55	0.61 ± 0.92	1.87 ± 1.73	0.11 ± 0.29

#### 3.2.3 Comparisons of lateral profiles for different field sizes

**FIGURE 5:**
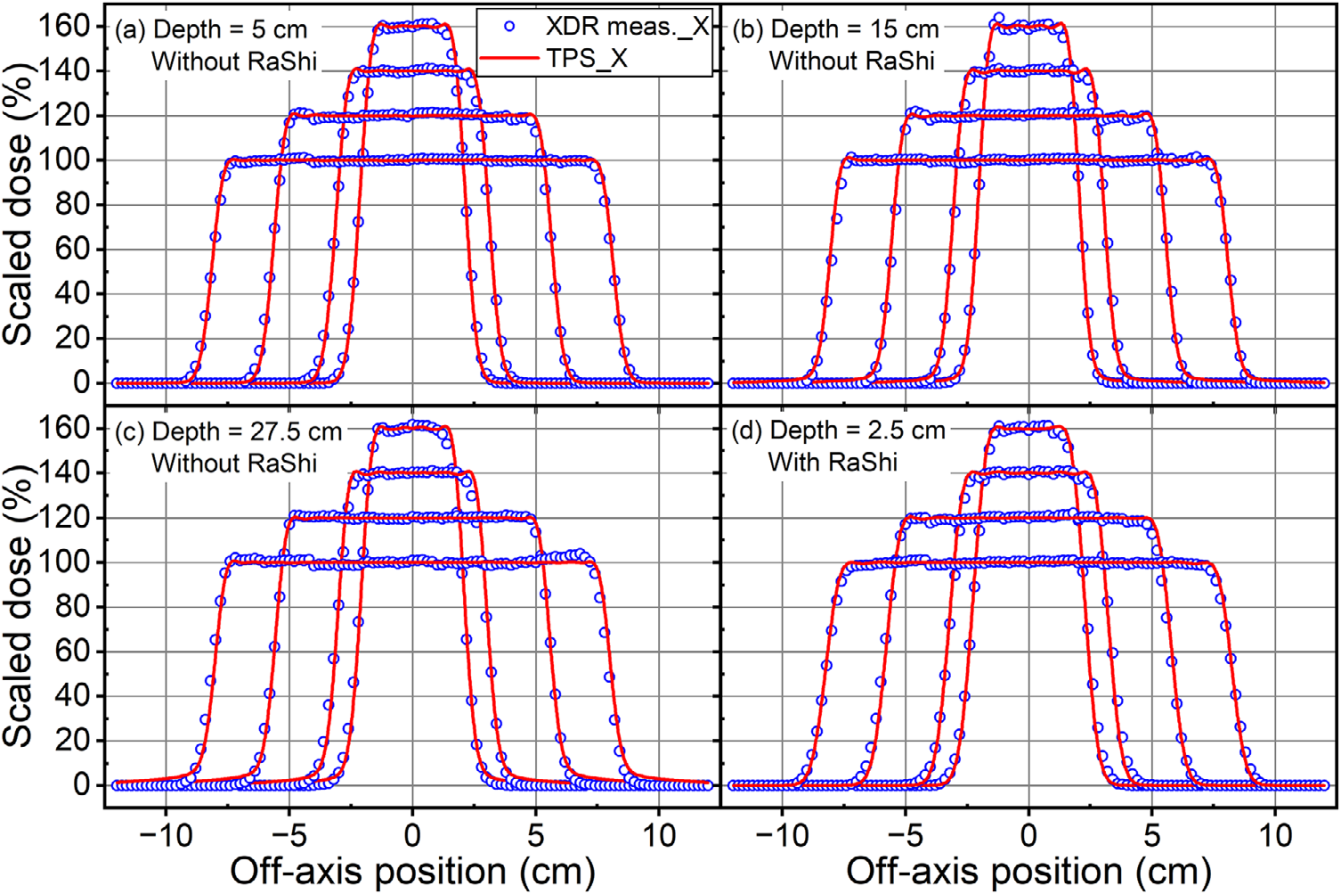
Comparisons between RS calculated and measured lateral dose profiles in X direction of different sizes of fields at depth of (a) 5.0 cm, (b) 15.0 cm, (c) 27.5 cm without the RaShi, and (d) 2.5 cm with the RaShi. Profiles were scaled by a factor of 1.6, 1.4, 1.2 and 1.0 for field size of 3.0 × 3.0, 5.0 × 5.0, 10.0 × 10.0, and 15.0 × 15.0 cm^2^, respectively.

The TPS‐calculated doses were compared with the measured lateral dose profiles in X direction using the XDR 729 detector for SOBP plans at various depths, with and without the RaShi, as shown in Figure 5. Overall, the target regions exhibited good agreement. However, discrepancies were observed, particularly at the right‐side edge of the field, where the measurements were 4% to 5% lower than the TPS calculations. Due to limitations of the 729 XDR, accurate measurement of out‐of‐field dose was not feasible, with the lowest measurement range at 0.1 Gy. The lateral profiles in both X and Y directions of a 10.0 × 10.0 cm^2^ field at 27.5 cm depth were measured and plotted in Supplement 4 to show the impact of asymmetry of high energy spots.

Table 2 lists the differences in parameters for evaluating field size in the SOBP plans. The TPS calculations demonstrated favorable agreement with the measurements, with nearly identical FWHM and other parameters differing by no more than 0.5 cm. However, a trend was observed wherein all TPS‐calculated penumbras were smaller than those measured, while the full width at 95% was larger in the TPS calculations compared to the measurements.

**TABLE 2:** Comparisons of parameters for field size presented in Figure 5.

			Deviations between TPS and means (cm)		
RaShi	Depth (cm)	Field size (cm^2^)	Penumbra	FWHM	Full width at 95%
No	5.0	3.0 × 3.0	−0.09	0.00	0.24
		5.0 × 5.0	−0.10	0.00	0.29
		10.0 × 10.0	−0.12	−0.01	0.26
		15.0 × 15.0	−0.11	0.01	0.25
No	15.0	3.0 × 3.0	−0.11	0.00	0.23
		5.0 × 5.0	−0.11	0.01	0.27
		10.0 × 10.0	−0.08	−0.01	0.20
		15.0 × 15.0	−0.12	0.03	0.27
No	27.5	3.0 × 3.0	−0.10	−0.01	0.24
		5.0 × 5.0	−0.18	0.00	0.42
		10.0 × 10.0	−0.11	−0.02	0.15
		15.0 × 15.0	−0.08	−0.01	0.11
Yes	2.5	3.0 × 3.0	−0.12	−0.01	0.22
		5.0 × 5.0	−0.13	−0.02	0.33
		10.0 × 10.0	−0.13	−0.01	0.33
		15.0 × 15.0	−0.12	0.00	0.34

#### 3.2.4 Absolute dose verification in shallow treatment targets

**FIGURE 6:**
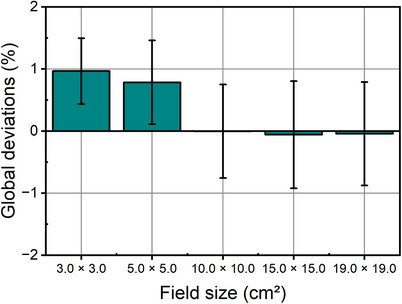
Global deviations between RS calculated and measured dose in five shallow targets with different field sizes. Five cuboid targets were 3.0 cm thick and centered at 2.7 cm depth. Error bars indicate standard deviations within analyzed PPCs.

Figure 6 shows the mean global dose deviations within five targets at shallow depth. RS calculated about 1.0% and 0.8% higher absolute dose for 3.0 × 3.0 and 5.0 × 5.0 cm^2^ field sizes than measurements, while for field sizes larger than 5.0 × 5.0 cm^2^, the deviation between RS calculation and measurements was almost zero.

#### 3.2.5 Comparisons in wedge and anthropomorphic phantom

**FIGURE 7:**
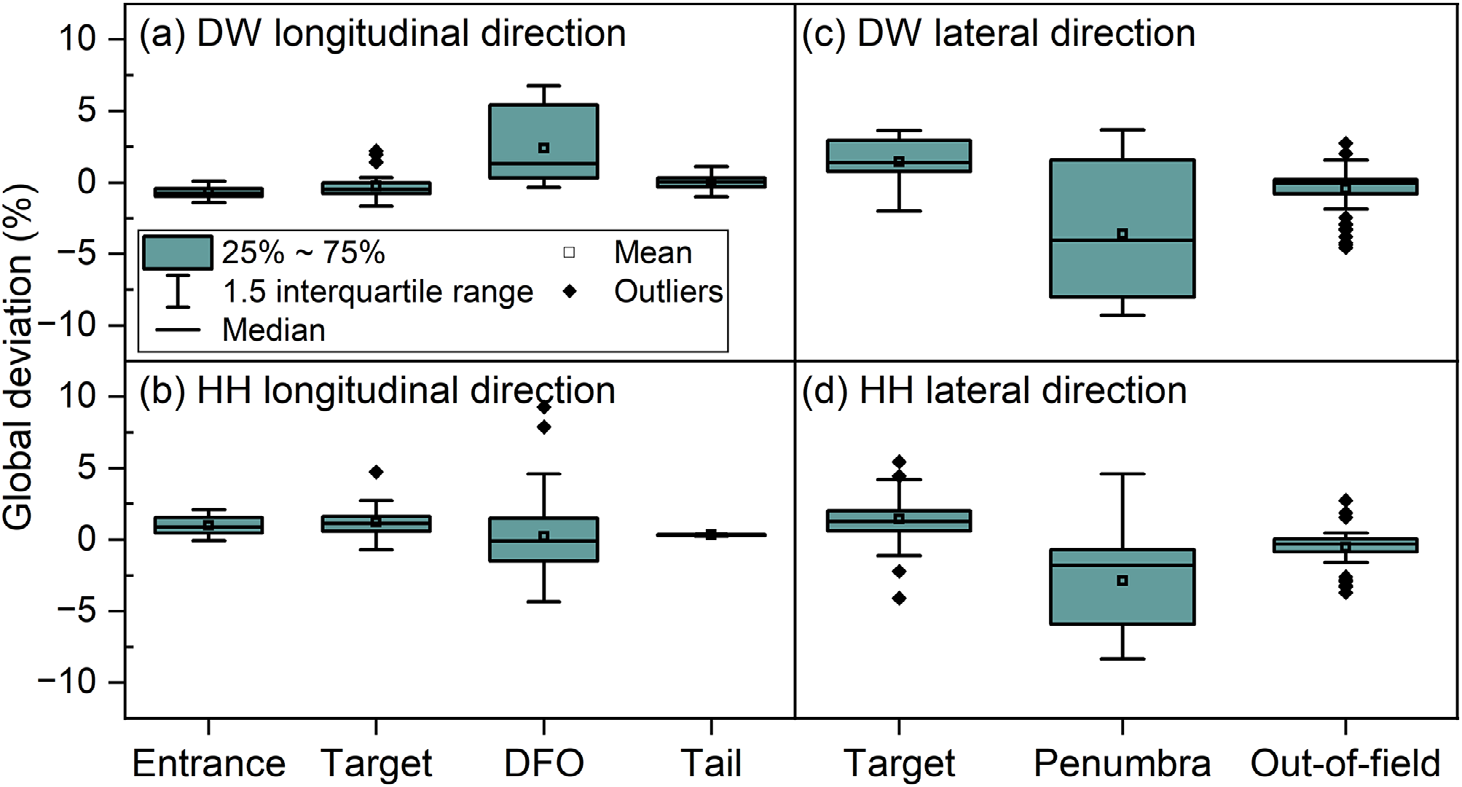
Global deviations between RS calculated and measured doses of the DW and HH plan.

Figure 7 presents the comparison between RS TPS calculated and measured doses of the DW plan and the HH plan. The results indicate that the mean global deviations are below 2% in the low‐gradient region of the entrance, target, tail, and out of field areas, and 4% in the high‐gradient regions of DFO and penumbra. Additionally, 1D gamma analysis (3%, 2 mm) was performed for the dose points in the penumbra and DFO, results revealing that all points met the criteria. However, larger deviations were observed in target region of the lateral direction, with most of points exhibiting large discrepancies at the field edges, where the maximum dose deviation reached 5.2%.

#### 3.2.6 Comparisons of RBE‐weighted dose in homogeneous phantom

**FIGURE 8:**
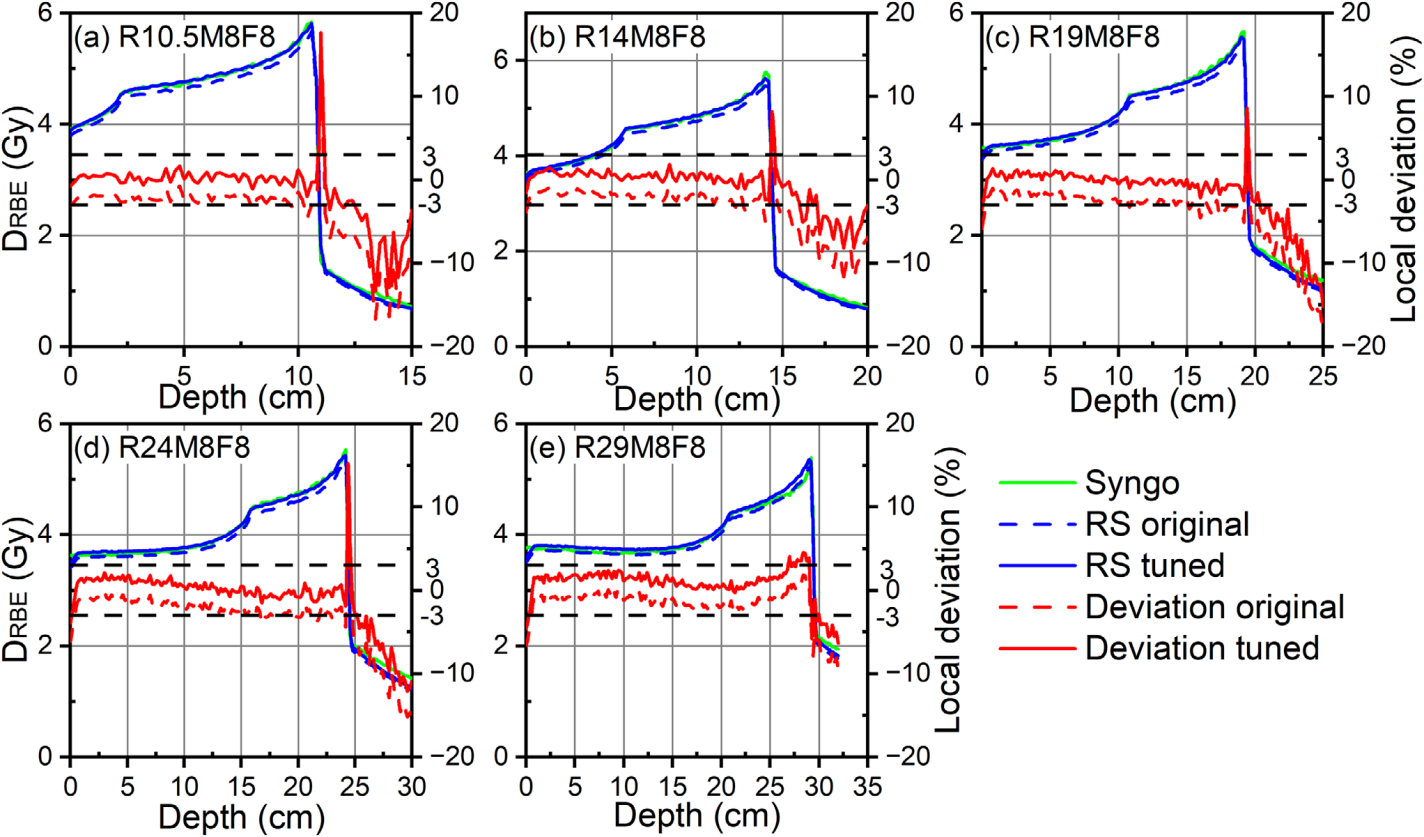
The comparisons of RBE‐weighted dose between RS and Syngo TPS calculations before and after RS RBE model tuning of five cubic plans R10.5M8F8, R14M8F8, R19M8F8, R24M8F8, and R29M8F8. Plans are labeled according to their Range in cm, Modulation width (SOBP) in cm, and square Field size in cm.

Figure 8 depicts the comparisons of RBE‐weighted dose of five cubic plans between RS and Syngo TPS calculations before and after refinement of the RS RBE model. Before the refinement, the maximum dose deviation in the homogeneous phantom reached 4% prior to the DFO. To address these discrepancies, we applied an average increase of 2.3% to the RBE_max_. Following the refinement process, the deviation decreased to less than 3% for the first four SOBPs. However, the SOBP targeting the deepest region exhibited differences still exceeding 3%, reaching up to 5%. These discrepancies corresponded to energies exceeding 408.07 Mev/u, where larger deviations in LETd were noted (refer to Section 3.1.3).

### 3.3 Patient case verification

#### 3.3.1 RBE‐weighted dose verification

**FIGURE 9:**
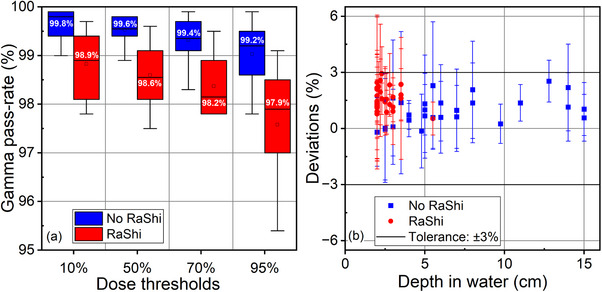
(a) Gamma pass‐rate of RBE‐weighted dose calculated by RS against Syngo. (b) PSQA results of 58 beams plotted against PPC array depth in water. Error bars indicate the standard deviations within analyzed PPCs of each beam.

In this study, we analyzed the RBE‐weighted dose distribution for a total of 58 beams from 20 patient plans. As depicted in Figure 9a, the gamma pass rate (3%, 3 mm) for the RBE‐weighted dose comparison between the RS and Syngo is greater than 97% for plans without the RaShi. For plans with the RaShi, although slightly lower, the pass rate remained greater than 95%.

#### 3.3.2 PSQA

Figure 9b presents PSQA results of 1366 PPC positions for 58 beams against PPC array depth in water. The PPC array depth was defined as the depth of the first row of PPCs. The mean global deviation of absorbed dose between TPS calculations and measurements was within 3% for all beams. Table 3 lists the statistics on analysis of PSQA for treatment plans. The mean global deviation is higher for the beams with the Rashi (1.60%) than those without the RaShi (0.96%). Furthermore, if we compare beams with and without the RaShi at a depth less than 5.5 cm (which was the maximum depth of PPC array of beams with the RaShi), the mean deviation without the RaShi was 0.70%. Additionally, the 1D gamma pass‐rate (3%, 3 mm) was above 90% for all beams.

**TABLE 3:** Statistics on analysis of PSQA for treatment plans.

		No RaShi	With RaShi
Measured PPC No.	Included	636	730
	Gamma passed	631	719
	Overall Pass‐rate (%)	99.2	98.5
PPC array depth (cm)	Median	5.5	2.2
	Min.	2.0	2.0
	Max.	15.0	5.5
Deviations (%)	Mean	0.96	1.60
	Min.	−0.19	0.53
	Max.	2.53	2.93

## 4 DISCUSSION

In this study, we performed a comprehensive commissioning of the RS TPS for scanned CIRT. Throughout this process, we verified not only the absorbed dose but also the RBE‐weighted dose. By carefully modeling the beam, we observed good agreement between the calculated absorbed dose and measurements, while the RBE‐weighted dose closely matched results from the clinically used TPS, except for the high‐energy part. Furthermore, this investigation provided insights into the limitations of the current carbon‐ion beam PBA and modeling.

During the absorbed dose commissioning, while overall mean dose discrepancies were within 3%, relatively higher deviations persisted in plans at the lowest depth and involving the Rashi, with a remaining 1% higher deviation impacting both absorbed and subsequently RBE‐weighted dose calculations. This finding is consistent with previous studies.^6^ Several reasons might contribute. First, absolute dose calibration performed at the entrance of the Bragg peak, introducing uncertainties due to measurements at the climbing part of the peak of lower energies. Second, the RS PBA has limitations, particularly in accounting for secondary particles and nuclear halo in the RaShi and their subsequent transport in air. A significant air gap (112.6 cm) exists between the beam exit and the isocenter, with over 30 cm between the RaShi and the patient for safety. These algorithm limitations and the long air gap notably affect dose calculation accuracy, resulting in calculations 1% higher than measurements in plans with the RaShi, even after adjustment. Proton PBAs also have shortage in terms of dose calculation accuracy when a RaShi and large air gap are used, but the development of proton MC algorithms improves accuracy.^31^, ^32^, ^33^ Our findings suggest that developing a clinical carbon‐ion MC algorithm is necessary for enhancing dose calculation accuracy in CIRT.

For the beam model generation, the Bragg peak and its tail were modeled based on the measurements, considering finite volume of the Bragg peak chamber and the WEL for the composition of nozzle and air gap. The calculated ranges and dose distributions agreed well with measurements in both DDDs and SOBPs. However, when comparing the lateral profile, the local absorbed dose differences exceeded 5% at field edges in both homogeneity and heterogeneity cases due to spot profile discrepancies between TPS beam model and reality. The RS TPS approximates lateral spot profile shape in air with Gaussian and allows only symmetrical simulation in X and Y. However, the spots in reality are asymmetric in the X direction, and the asymmetry increases with energy. The dose calculation in the target is less sensitive to the spot profile changes than at the target's edge and penumbra. In this study, spot size varied by 10%, much less than reported 25%,^34^ and did not obviously deviate the dose within the target. Spot and grid ratio higher than four still yielded relatively homogeneous dose distribution within treatment field. However, the dose variations at the field edge and in the penumbra corresponded with the asymmetrical shape of the spot profile, resulting in relatively larger discrepancies. This limitation affected dose calculations in homogenous WP, DW, and HH phantom, similar to findings reported previously.^35^ The treatment plans at our center are mostly designed employing the IMPT strategy with multiple beam angles by rotating the treatment table, which allows the discrepancies at the edge of the treatment target to be smeared out by modulated beam intensity and different beam angles.

The performance of RS on dose calculation for shallow targets was evaluated for field sizes ranging from 3.0 × 3.0 cm^2^ up to almost the largest field size available in our particle therapy system. The modulation width 3.0 cm and center depth 2.7 cm were chosen to make sure the lowest energy was used in plan optimization and a uniform dose distribution in the target was achieved without the use of a RaShi. Results showed that RS calculated 0.8%–1.0% higher dose than measurements for field size no larger than 5.0 × 5.0 cm^2^, indicating that RS overestimated dose in shallow and small targets. The study on small fields of low energy proton beams has also shown the overestimation of TPS.^36^ The reason of this disagreement is that the nuclear halo effect is not precisely modeled for small and shallow targets in the TPS. However, the magnitude of disagreement over output of small and shallow fields is lower for carbon‐ion beams than for proton beams, since the carbon‐ion beams are less laterally scattered in water than proton beams.

In lateral heterogeneities, the carbon dose engine employs a single pencil beam per spot, unlike the proton model, which decomposes spot fluence into 19 sub‐pencil beams. In HH and DW phantoms, TPS calculations matched well with measurements in the target and entrance regions, possibly due to heavier carbon particles causing less scattering. RS pencil beam algorithms for carbon‐ion beams can effectively handle heterogeneities and oblique beam angles.

During the transportation process, carbon ions undergo additional nuclear reactions with the surrounding medium, leading to the generation of a substantial out‐of‐field low‐dose volume laterally. Incorporating this phenomenon into the modeling is crucial, as it results in the significant reduction of dose within the central Gaussian region. Measurement of the out‐of‐field area was conducted using a HH and irregular entrance shape of a DW phantom equipped with pinpoint chambers. The observed mean global deviations were found to be within 1%. When taking a closer look at the point doses in the out‐of‐field region, points with dose deviation larger than 3% had a DTA smaller than 1 mm, since all of these points lay in 10%–20% dose region where the dose gradient was larger than 0.1 Gy/mm. The dose deviations in region where doses were less than 10% of the PD were all within 1%. These results indicate that RS's modeling of the nuclear halo is clinically acceptable even when there is inhomogeneity and oblique incidence.

In CIRT, RBE model commissioning is crucial yet rarely reported. Initially, we observed a significant 3%–4% variation in RBE‐weighted dose between the clinical TPS and RS TPS, despite minimal differences in absorbed dose (within 1%). This discrepancy primarily stemmed from variations in the RBE factor, where small differences in the TPS RBE model led to variations in calculated absorbed dose during optimization. These differences originated from the different approaches that RS and Syngo use to calculate RBE‐weighted dose. RS uses the simulated fragment spectra to compute the RBE factor, then multiply the absorbed dose computed from measured IDDs to compute RBE‐weighted dose, while Syngo uses FLUKA simulated IDDs to compute RBE‐weighted dose. The difference between FLUKA simulated and measured IDDs is around 2% at the entrance of high energies. LET comparison facilitated quantifying these differences for energies higher than 408.07MeV/u, which corresponds to depths exceeding 28.5 cm. These energies are predominantly employed in one of the three beams for treating pancreatic cancer from the superior direction at the back. However, the RBE discrepancies were mitigated when considering all three beams collectively, and the gamma analysis showed a high pass rate for abdominal cases even using these high energies.

There are pronounced disparities also observed in the peak region and the tail region when comparing LETds between RS and Syngo calculations. The former is likely attributed to the interpolation of beam parameters from a discrete set of beam energies and fragment spectra. The discrepancies in the composition of fragment spectra, especially for ion types with Z = 4 and 5, can be explained for the tail region. Notably, these discrepancies do not appear to significantly affect the RBE‐weighted dose.^8^ This correction aligns with the principle that radiation should induce the same biological effect with consistent energy, dose, and delivery methods.^37^ Our study highlights the importance of validating fragment spectra baseline accuracy for RBE‐weighted dose calculations by TPS, with the LETd comparison method serving as a valuable tool in this regard.

A limitation of this study is that the performance of RS TPS for field size smaller than 3.0 ×3.0 cm^2^ was not evaluated. Study on proton small field size revealed that pencil beam modeling of the lateral dose distribution of the TPS could be the severe limitation in dose calculation accuracy for the smaller and shallower targets,^38^ while double Gaussian beam modeling and MC‐based dose engine of RS can significantly improve dose calculations in small proton irradiation fields.^39^ The RS performance on small carbon‐ion fields was not included in this study because of the lack of small volume high‐resolution radiation detectors, which are crucial for absolute measurements in small carbon‐ion fields. In our facility every particle beam treatment plan must be verified through a standard PSQA procedure, either by measurements in the WP^30^ or comparison against a fast MC dose engine^40^ to ensure dose accuracy before treating patients. Further investigation is warranted to assess the efficacy of treatment planning for very small targets in clinical practice.

## 5 CONCLUSION

The RS TPS with PBAs for CIRT has been commissioned in this study. The absorbed doses calculated by the RS TPS for both regular‐shaped plans and patient plans demonstrated good agreement with measurements in water, although slightly higher discrepancies observed at the penumbra and in plans involving the RaShi. The accuracy of RBE‐weighted dose calculation was assessed by comparing the RS calculated RBE‐weighted doses with those computed by the clinical Syngo TPS. Based on the results reported in our study, the PBA of RS TPS V10B for carbon‐ion dose calculation is adequate for treatment planning of most clinical cases. However, improvements in fragment spectra and further investigation of small field sizes are needed for the future.

## AUTHOR CONTRIBUTIONS

Wei Sun and Weiwei Wang contributed equally to this work and should be regarded as co‐first authors. Wei Sun generated the beam plans, performed measurements, analyzed the data, and drafted the manuscript, and Weiwei Wang generated the beam plans, performed measurements, tuned the beam model, and revised the manuscript. Zhijie Huang performed measurements. Jingfang Zhao helped data collection and assisted in drafting the manuscript. All authors read and approved the final manuscript.

## CONFLICT OF INTEREST STATEMENT

The authors declare no conflicts of interest.

Stefanie Käss from RaySearch Laboratories helped with part of the measurements and beam modeling process.

## Supporting information



Supplementary Information
