# Supplementary material for: Commissioning of a commercial treatment planning system for scanned carbon‐ion radiotherapy
Source: J Appl Clin Med Phys. 2024 Nov 29;26(3):e14580. doi: 10.1002/acm2.14580 (PMC11905250; doi:10.1002/acm2.14580)
Supplement: Supplementary file 1 — Supplementary Information [file ACM2-26-e14580-s001.docx]

**Supplement 1** Overview of the measurement setups.

| Measurement items | Equipment used |
| --- | --- |
| 1. Lateral spot profiles in air | Extended dose range (EDR2) films (Carestream) |
| 1. Beam range in water | PEAKFINDER (T41030, PTW) with a Bragg peak chamber (T34080, PTW) as measuring chamber |
| 1. Absolute output calibration | Advanced Markus ionization chamber (T34045, PTW) in a MP3-P water phantom |
| 1. Absolute dose verification in spread-out Bragg peaks | 24 PinPoint ionization chambers (T31015, PTW) in a MP3-P water phantom |
| 1. Depth doses in the homogeneous condition | 24 PinPoint ionization chambers (T31015, PTW) in a MP3-P water phantom |
| 1. Lateral profiles for different field sizes | OCTAVIUS Detector 729 XDR (T10042, PTW) with solidwater phantoms |
| 1. Comparisons in wedge and anthropomorphic phantom | 24 PinPoint ionization chambers (T31015, PTW) in a MP3-P water phantom |
| 1. Patient-specific quality assurance | 24 PinPoint ionization chambers (T31015, PTW) in a MP3-P water phantom |

**Supplement 2** Overall statistics of evaluated treatment plans. Numbers in brackets are beam numbers.

|  |  | No RaShi | RaShi |
| --- | --- | --- | --- |
| Case No. by target site | Head & Neck | 3 (9) | 3 (10) |
|  | Thorax | 3 (9) | 2 (6) |
|  | Abdomen | 2 (5) | - |
|  | Pelvis | 2 (4) | 1 (4) |
|  | Breast | - | 1 (2) |
|  | Extremities | - | 3 (9) |
|  | Total | 10 (27) | 10 (31) |

**Supplement 3** The comparisons of depth dose distributions between RS calculated and measured values.





**Supplement 4** Measured profiles in X and Y directions of a 10.0 × 10.0 cm2 field at 27.5 cm depth.
